# Treatment choice and sexual health outcomes in gay and bisexual men with prostate cancer

**DOI:** 10.1038/s41391-025-01007-1

**Published:** 2025-08-06

**Authors:** Daniel R. Dickstein, Alex J. Bates, Christopher W. Wheldon, Ryan Haggart, Kristine M. C. Talley, Deborah C. Marshall, B. R. Simon Rosser

**Affiliations:** 1 Department of Radiation Oncology, Icahn School of Medicine at Mount Sinai, New York, NY, USA.; 2 Division of Epidemiology and Community Health, University of Minnesota, School of Public Health, Minneapolis, MN, USA.; 3 Department of Social and Behavioral Sciences, Temple University, College of Public Health, Philadelphia, PA, USA.; 4 Department of Urology, University of Minnesota, Minneapolis, MN, USA.; 5 Adult and Gerontological Health, University of Minnesota, School of Nursing, Minneapolis, MN, USA.

## Abstract

**BACKGROUND:**

The differential impacts of radical prostatectomy (RP) and external beam radiotherapy (EBRT) on sexual health in gay and bisexual men (GBM) with prostate cancer remain understudied, limiting recommendations incorporating preferred sexual behavior.

**METHODS:**

Secondary analysis of Restore-2 compared outcomes in GBM after RP (*n* = 127) or EBRT (*n* = 27).

**RESULTS:**

Compared to RP, EBRT was associated with lower rates of climacturia (11% vs. 59%, *p* < 0.001), condom-related erectile dysfunction (27% vs. 51%, *p* = 0.047), and decreased penile length/girth (49% vs. 69%, *p* = 0.07), but higher rates of anodyspareunia (33% vs. 14%, *p* = 0.06).

**CONCLUSIONS:**

Exploratory findings suggest EBRT for insertive intercourse, RP for receptive intercourse.

## INTRODUCTION

Early-stage prostate cancer can be treated with external beam radiotherapy (EBRT), radical prostatectomy (RP), brachytherapy, or active surveillance [ref. 1]. Consequently, research has characterized treatment impacts on quality of life outcomes, including sexual health outcomes [ref. 2]. However, research on the sexual effects of prostate cancer has been limited to studying the impacts on erections stron enough for vaginal intercourse, producing a heterocentric bias in the literature and overlooking gay and bisexual men (GBM) with prostate cancer [ref. 2]—representing approximately 31,000 new annual diagnoses in the United States [ref. 3].

Sexual health considerations differ between GBM and cisgender heterosexual men as well as among GBM themselves [ref. 2–4]. Compared to heterosexual men, GBM more commonly engage in insertive anal intercourse (requiring 33% greater erectile firmness than vaginal intercourse), receptive anal intercourse (RAI, where the prostate facilitates pleasure [ref. 4]), and oral intercourse [ref. 2]. Among GBM, many prefer specific behaviors and identify with roles-in-sex: “top” (insertive anal intercourse), “bottom” (RAI), “versatile” or “vers” (both), or “side” (neither; does not engage in anal intercourse, preferring sexual behaviors such as oral intercourse and mutual masturbation) [ref. 2, 5].

Individual sexual behavior preferences may create distinct treatment recommendations [ref. 5]. Those who prioritize insertive intercourse may be particularly concerned with erectile function and penile size; those who prioritize oral sex may be more affected by climacturia and arousal incontinence; and those who prioritize RAI may be most concerned with mitigating pain and preserving pleasure [ref. 2, 5]. However, little is known about how RP and EBRT differentially affect these sexual health outcomes in GBM. To provide guidance during treatment discussions, this study compared sexual health outcomes of GBM with early-stage prostate cancer treated with RP or EBRT.

## METHODS

*Restore-2* (NCT03343093) was a randomized prospective clinical trial investigating a sexual health rehabilitation program for GBM prostate cancer survivors [ref. 6]. Methods have been detailed previously [ref. 7], and our Institutional Review Board approved this study [ref. 6]

For this secondary analysis, we included only those who self-reported treatment with RP or EBRT without androgen deprivation therapy for early-stage prostate cancer, defined as prostate specific antigen ≤ 20; grade group 1, 2, 3; stage 1, 2. Since prospective randomized controlled trials demonstrate that radiation-induced toxicities—including sexual, bowel, and urinary dysfunction—plateau by 24 months, we used a ≥ 2-year minimum follow-up window for fair treatment comparisons [ref. 8, 9].

Sexual health outcomes were assessed using the Sexual Minorities and Prostate Cancer Scale (SMACS) [ref. 10]. Logistic and linear regression were used to estimate associations between treatment choice and SMACS dichotomized individual outcomes and multidimensional subscales, respectively. Models were adjusted for age, comorbidity count, medications, and time since treatment. Outcomes were measured after treatment began rather than at true baseline; therefore, baseline toxicities were not included as covariates as they could lie on the causal pathway.

Post-hoc power analyses determined adequate power to detect risk differences between treatment groups ranging from 0.24 (for orgasm change) to 0.31 (for decreased pleasure during RAI) for dichotomous outcomes, and from 0.31 (for urinary incontinence in sex) to 0.78 (for problematic RAI) points for continuous SMACS outcomes. We considered *p* < 0.05 as significant and *p* < 0.1 as evidence of a trend. Results should be interpreted as exploratory.

## RESULTS

Of the 401 participants in the parent study, 247 were excluded (117 did not complete the questionnaire ≥ 2 years post-treatment, 85 underwent multiple treatments, 32 had advanced-stage disease,12 had indeterminate-stage disease, 1 underwent brachytherapy). Thus, 154 met inclusion criteria (127 treated with RP, 27 treated with EBRT). Participants had a mean age of 65 (standard deviation [SD]: 7) years and completed treatment 6.7 (SD: 4.8) years prior. RP-treated participants were significantly younger, took fewer medications adversely affecting sexual health, and were further removed from treatment at the time of questionnaire completion compared to EBRT-treated participants (Table 1).

EBRT-treated participants compared to RP-treated participants were less likely to report climacturia (11% vs. 59%, *p* < 0.001), difficulty with condoms due to erectile dysfunction (27% vs. 51%, *p* = 0.047), decreased penile length/girth (49% vs. 69%, *p* = 0.07), and more likely to report anodyspareunia (painful RAI; 33% vs. 14%, *p* = 0.06). On multidimensional subscales, GBM treated with EBRT reported better quality of life related to urinary incontinence during sex (Table 1).

## DISCUSSION

This is the first study to compare sexual health outcomes after RP or EBRT in GBM with prostate cancer [ref. 5]. Consistent with studies in the general prostate cancer population, treatment-related morbidity differed by treatment modality [ref. 1].

GBM who prioritize insertive intercourse (anal, vaginal, and oral) may experience better sexual health outcomes with EBRT compared to RP, while those who prioritize RAI may experience fewer anal pain-related complications with RP. However, findings warrant further investigation given sample size differences (EBRT = 27 vs. RP = 127).

For RAI, EBRT may be associated with anodyspareunia compared to RP; however, no differences were observed in dichotomized RAI sexual pleasure or composite problematic RAI scores between treatments. Future research should further characterize how EBRT and RP may differentially impact individual RAI outcomes. Previous RAI research demonstrated brachytherapy, compared to EBRT, may be associated with less pain, potentially due to more precise targeting that spares surrounding tissues, but also less pleasure, as more targeted treatments directly affect the prostate [ref. 3]. While EBRT directly damages the prostate and may indirectly damage surrounding structures (e.g. the sensate anus), RP removes the entire prostate [ref. 3]. Better characterizing treatment effects on pain and pleasure during RAI can allow clinicians to tailor recommendations for mitigating pain or preserving pleasure.

Our study contains several limitations. First, the exploratory nature, unbalanced and small sample size, and enrollment criteria requiring existing baseline sexual/urinary problems introduce selection bias and limit estimate precision. Second, we could not control for baseline toxicities, including pre-existing RAI difficulties, as these measures were not collected in the parent study, creating potential residual confounding. Third, the 2-year post-treatment assessment introduces recall bias. Fourth, our sample was demographically homogeneous (predominantly White, well-educated, English-speaking, gay, cisgender men), limiting generalizability to the experiences of GBM survivors who are non-white, lower socio-economic status, or less connected to healthcare, who may experience greater disparities. Finally, a *p*-value threshold <0.1 does not guarantee a statistically significant difference with a larger sample size; results should be interpreted cautiously as exploratory findings.

To fully understand the association between prostate cancer treatments and sexual health outcomes in GBM, longitudinal prospective studies or larger sample sizes with a more demographically diverse cohort are necessary. Meanwhile, these exploratory findings provide the first evidence-based guidance for RP versus EBRT shared-decision-making conversations with GBM with prostate cancer. After inquiring about sexual orientation, gender identity, sexual behaviors, and preferred role-in-sex, clinicians should begin to incorporate preferences into treatment recommendations.

## Supplementary Material

Supplementary Table 1

**Supplementary information** The online version contains supplementary material available at https://doi.org/10.1038/s41391-025-01007-1.

## Figures and Tables

**Table 1.** Association between treatment choice and sexual health outcomes in gay and bisexual men treated for early-stage prostate cancer in unadjusted and adjusted regression models. Reference group: radical prostatectomy.

Sexual Health Outcomes ^a^	Unadjusted			Adjusted ^b^		
Individual SMACS outcomes^c^	Predicted Risk [95% CI]	Odds Ratio [95% CI]	*p*-value^d^	Predicted Risk [95% CI]	Odds Ratio [95% CI]	*p*-value^d^
Climacturia^e^		0.09 [0.03, 0.32]	<0.001		0.08 [0.02, 0.31]	<0.001
Radical prostatectomy	59.1 [50.5, 67.6]			58.7 [50.0, 67.3]		
External beam radiotherapy	11.5 [0, 23.8]			11.0 [0, 23.1]		
Lack of sexual desire		0.75 [0.32, 1.79]	0.52		0.85 [0.32, 2.27]	0.75
Radical prostatectomy	69.3 [61.3, 77.3]			68.4 [60.2, 76.6]		
External beam radiotherapy	63.0 [44.7, 81.2]			65.0 [46.0, 84.0]		
Lack of sexual confidence		0.97 [0.30, 3.12]	0.96		0.67 [0.18, 2.51]	0.56
Radical prostatectomy	85.0 [78.8, 91.2]			85.5 [79.5, 91.5]		
External beam radiotherapy	84.6 [70.7, 98.5]			80.2 [62.3, 98.0]		
Erectile dysfunction		0.95 [0.29, 3.07]	0.93		0.68 [0.18, 2.54]	0.57
Radical prostatectomy	85.8 [79.8, 91.9]			86.2 [80.4, 92.1]		
External beam radiotherapy	85.2 [71.8, 98.6]			81.4 [64.7, 98.0]		
Not using a condom due to difficulty keeping an erection		0.54 [0.21, 1.38]	0.20		0.34 [0.12, 0.99]	0.047
Radical prostatectomy	49.6 [40.4, 58.7]			50.9 [41.9, 60.0]		
External beam radiotherapy	34.8 [15.3, 54.2]			27.1 [9.3, 44.8]		
Anejaculation		2.38 [0.52, 10.83]	0.26		1.89 [0.38, 9.50]	0.44
Radical prostatectomy	83.5 [77.0, 89.9]			83.8 [77.6, 90.0]		
External beam radiotherapy	92.3 [82.1, 100]			90.5 [77.8, 100]		
Anodyspareunia^f^		3.27 [1.18, 9.04]	0.02		3.28 [0.98, 11.0]	0.06
Radical prostatectomy	14.0 [7.4, 20.6]			14.3 [7.7, 20.9]		
External beam radiotherapy	34.8 [15.3, 54.2]			33.1 [12.9, 53.4]		
Decreased pleasure during receptive anal intercourse		1.53 [0.62, 3.76]	0.35		0.93 [0.33, 2.68]	0.90
Radical prostatectomy	35.6 [26.4, 44.8]			38.1 [28.9, 47.3]		
External beam radiotherapy	45.8 [25.9, 65.8]			36.7 [17.4, 56.0]		
Decreased penile length/girth		0.45 [0.19, 1.06]	0.07		0.41 [0.15, 1.09]	0.07
Radical prostatectomy	69.0 [61.0, 77.1]			68.7 [60.7, 76.7]		
External beam radiotherapy	50.0 [30.8, 69.2]			48.6 [28.3, 68.9]		
Orgasm change		2.05 [0.72, 5.8]	0.18		1.82 [0.60, 5.57]	0.29
Radical prostatectomy	68.3 [60.1, 76.4]			68.3 [60.1, 76.5]		
External beam radiotherapy	81.5 [66.8, 96.1]			79.6 [63.0, 96.1]		
Multidimensional SMACS outcomes (Range: 1 to 5)^g^	Predicted Value [95% CI]	Mean Difference [95% CI]	*p*-value^h^	Predicted Value [95% CI]	Mean Difference [95% CI]	*p*-value^h^
Sexual satisfaction		0.13 [−0.32, 0.59]	0.56		0.25 [−0.24, 0.74]	0.32
Radical prostatectomy	2.36 [2.17, 2.55]			2.35 [2.15, 2.54]		
External beam radiotherapy	2.50 [2.08, 2.91]			2.59 [2.16, 3.03]		
Sexual confidence		0.31 [−0.19, 0.81]	0.23		0.43 [−0.11, 0.98]	0.11
Radical prostatectomy	3.23 [3.02, 3.44]			3.21 [3.00, 3.42]		
External beam radiotherapy	3.54 [3.08, 4.00]			3.65 [3.16, 4.13]		
Frequency of sexual problems		0.25 [−0.21, 0.70]	0.28		0.29 [−0.20, 0.79]	0.25
Radical prostatectomy	3.43 [3.24, 3.62]			3.42 [3.22, 3.61]		
External beam radiotherapy	3.68 [3.27, 4.09]			3.71 [3.27, 4.15]		
Sexual distress^i^		0.24 [−0.18, 0.66]	0.27		0.33 [−0.13, 0.79]	0.16
Radical prostatectomy	2.96 [2.79, 3.14]			2.95 [2.77, 3.13]		
External beam radiotherapy	3.20 [2.82, 3.59]			3.28 [2.87, 3.69]		
Urinary incontinence in sex^j^		0.91 [0.51, 1.30]	<0.001		0.92 [0.50, 1.34]	<0.001
Radical prostatectomy	4.01 [3.85, 4.17]			4.02 [3.86, 4.19]		
External beam radiotherapy	4.92 [4.55, 5.28]			4.94 [4.57, 5.32]		
Problematic receptive anal intercourse^k^		−0.15 [−0.60, 0.29]	0.50		−0.13 [−0.61, 0.35]	0.60
Radical prostatectomy	4.49 [4.32, 4.65]			4.48 [4.30, 4.65]		
External beam radiotherapy	4.33 [3.92, 4.75]			4.35 [3.91, 4.79]		

*CI* Confidence Interval, *SMACS* Sexual Minorities and Prostate Cancer Scale.

a Sexual health outcome data was taken from the 24-month follow-up survey.

b Adjusted for comorbidities, age, medications adversely affecting sexual outcome, erectile dysfunction medications, time since treatment; Reference=Radical Prostatectomy.

c Individual SMACS outcomes were self-reported on a 5-point Likert scale on how often each has been a problem, and were dichotomized to either experiencing the problem to “none of the time” or “anytime”.

d Dichotomized individual sexual health outcomes were analyzed by treatment choice with logistic regression. Predicted probabilities and their 95% CI were bounded between 0 and 1 to reflect feasible values.

e Climacturia is orgasm associated urinary incontinence.

f Anodyspareunia is increased pain or bleeding during receptive anal intercourse.

g Multidimensional SMACS subscales scores are the mean of responses to each item in the subscale, with scores ranging from 0 to 5, with higher scores (i.e. those closer to 5) indicating better sexual health and/or less distress. Cronbach’s alpha coefficients range from 0.64 (frequency of sexual problems subscale) to 0.89 (urinary incontinence during sex subscale).

h Mean differences for SMACS subdomain scores were analyzed by treatment choice with linear regression.

i The mean of the sexual satisfaction, sexual confidence, and frequency of sexual problems items represents the sexual distress scale.

j Problematic receptive anal intercourse encompasses anodyspareunia, decreased pleasure, and issues with orgasm during receptive anal intercourse.

k Urinary incontinence during sex encompasses both arousal and orgasm (climacturia) associated incontinence.

## Data Availability

The raw data supporting the conclusions of this article can be made available by the authors, without undue reservation, upon request. At the end of study completion, all data will be available at the Open Inter-university Consortium for Political and Social Research (OpenICPSR) Data Repository.
