# Supplementary material for: Treatment choice and sexual health outcomes in gay and bisexual men with prostate cancer
Source: Prostate Cancer Prostatic Dis. Author manuscript; Available in PMC 2026 Jan 30. (PMC12854925; doi:10.1038/s41391-025-01007-1)
Supplement: Supplementary Table 1 [file NIHMS2112345-supplement-Supplementary_Table_1.docx]

| Supplementary Table 1: Demographics and Disease Characteristics in Gay and Bisexual Treated for Early-Stage Prostate Cancer | | | | |
| --- | --- | --- | --- | --- |
| Variable^a^ | Radical Prostatectomy (n=127) | External Beam Radiotherapy (n=27) | All Patients^b^ (n=154) | *p*-value^c^ |
| Age, years |  |  |  | 0.01 |
| Mean (SD) | 64.6 (6.5) | 68.4 (5.7) | 65.3 (6.5) |  |
| Race/ethnicity^d^, n (%) |  |  |  | 0.47 |
| White, non-Hispanic | 114 (90) | 26 (96) | 140 (91) |  |
| Non-White and/or Hispanic | 13 (10) | 1 (4) | 14 (9) |  |
| Education, n (%) |  |  |  | 0.60 |
| Less than bachelor’s degree | 27 (21) | 6 (22) | 33 (21) |  |
| Bachelor’s degree | 45 (35) | 12 (44) | 57 (37) |  |
| Graduate or professional degree | 55 (43) | 9 (33) | 64 (42) |  |
| Income, n (%) |  |  |  | 0.12 |
| <$35,000 | 19 (16) | 7 (30) | 26 (18) |  |
| $35,000-74,999 | 36 (29) | 8 (35) | 44 (30) |  |
| ≥$75,000 | 68 (55) | 8 (35) | 76 (52) |  |
| Relationship status, n (%) |  |  |  | 0.08 |
| Single, divorced, widowed | 64 (50) | 18 (69) | 82 (54) |  |
| Married or relationship | 63 (50) | 8 (31) | 71 (46) |  |
| Sexuality, n (%) |  |  |  | 0.99 |
| Gay/homosexual | 114 (90) | 25 (93) | 139 (90) |  |
| Bisexual | 13 (10) | 2 (7) | 15 (10) |  |
| Years since diagnosis |  |  |  | 0.07 |
| Mean (SD) | 7.7 (4.8) | 5.9 (3.4) | 7.4 (4.7) |  |
| Years since treatment |  |  |  | 0.04 |
| Mean (SD) | 7.0 (5.0) | 4.9 (3.2) | 6.6 (4.8) |  |
| Comorbidity count^e^ |  |  |  | 0.17 |
| Mean (SD) | 1.9 (1.5) | 2.4 (1.8) | 2.0 (1.6) |  |
| Medications adversely affecting sexual function^e^ |  |  |  | 0.03 |
| Mean (SD) | 1.0 (1.1) | 1.6 (1.5) | 1.1 (1.2) |  |
| Erectile dysfunction medication^f^, n (%) |  |  |  | 0.23 |
| Taking medication to help erectile   function | 68 (54) | 18 (67) | 86 (56) |  |
| Gleason score, grade group^g^, n (%) |  |  |  | 0.52 |
| 3+3, grade group 1 | 28 (30) | 8 (40) | 36 (32) |  |
| 3+4, grade group 2 | 40 (44) | 9 (45) | 49 (44) |  |
| 4+3, grade group 3 | 24 (26) | 3 (15) | 27 (24) |  |
| Stage^f^, n (%) |  |  |  | 0.26 |
| I | 57 (66) | 15 (79) | 72 (68) |  |
| II | 30 (35) | 4 (21) | 34 (32) |  |
| PSA^f^ range, n (%) |  |  |  | 0.75 |
| 0 ng/mL < PSA < 10 ng/mL | 93 (87) | 21 (84) | 114 (86) |  |
| 10 ng/mL < PSA < 20 ng/mL | 14 (13) | 4 (16) | 18 (14) |  |
| Abbreviations: SD, Standard Deviation; PSA, Prostate Specific Antigen.  ^a^ Participant self-reported demographic and clinical characteristics at diagnosis were taken from the baseline survey.  ^b^ The parent study recruited participants who were sexual (i.e., gay and bisexual men) and gender minorities, lived in the United States, received a prostate cancer diagnosis, were internet users, and self-reported a sexual and/or urinary problem. Transgender women were welcome to participate, although none enrolled.  ^c^ Characteristics were compared by treatment choice using *t*-tests for continuous variables and chi-square tests for categorical variables.  ^d^ Due to the small number of non-White and Hispanic participants, racial and ethnicity demographic information were collapsed into non-White and/or Hispanic or White and non-Hispanic.  ^e^ Information on comorbidities and medications collected are available in the parent trial protocol. Medications affecting sexual function included antidepressants, muscle relaxants, chemotherapy, hormone therapies, and medications for benign prostatic hyperplasia.  ^f^ Erectile dysfunction medications included sildenafil, vardenafil, tadalafil, avanafil, and an other option.  ^g^ Participants missing information on Gleason score/grade group, stage, and PSA were excluded (n=12). | | | | |
